# Molecular basis for CENP-N recognition of CENP-A nucleosome on the human kinetochore

**DOI:** 10.1038/cr.2018.13

**Published:** 2018-01-19

**Authors:** Tian Tian, Xiaorun Li, Yingying Liu, Chengliang Wang, Xing Liu, Guoqiang Bi, Xuan Zhang, Xuebiao Yao, Z Hong Zhou, Jianye Zang

**Affiliations:** 1 Hefei National Laboratory for Physical Sciences at Microscale, CAS Center for Excellence in Biomacromolecules, Collaborative Innovation Center of Chemistry for Life Sciences, and School of Life Sciences, University of Science and Technology of China, 96 Jinzhai Road, Hefei, Anhui 230026, China; 2 Key Laboratory of Structural Biology, Chinese Academy of Sciences, Hefei, Anhui 230027, China; 3 Department of Microbiology, Immunology & Molecular Genetics, University of California, Los Angeles, Los Angeles, CA, USA; 4 Anhui Key Laboratory for Cellular Dynamics & Chemical Biology, Hefei, Anhui 230027, China

## Dear Editor,

Recognition of CENP-A-containing chromatin by CENP-N is a critical step in the assembly of functional kinetochore at the centromere to enable accurate chromosome segregation during cell division^1,2^. CENP-N is recruited to centromeres during S phase and gradually dissociates during G2 phase. This dynamic assembly of CENP-N onto the centromere is regulated by the change in accessibility to CENP-A's RG loop (Arg80/Gly81) during the compacting-opening transition of centromeric chromatin structure through the cell cycle^3,4^. CENP-N has an N-terminal domain that binds to RG loop of CENP-A and a C-terminal domain that interacts with CENP-L to form CENP-N/CENP-L complex (CENP-LN), which further associates with CENP-C and CENP-HIKM to orchestrate kinetochore assembly^1,3,5,6^. Recent studies from hydrogen/deuterium exchange coupled to mass spectrometry (HXMS) have shown that nucleosomal DNA (−21 and −22 nt) had direct contacts with CENP-N in addition to CENP-A's RG loop^7^, suggesting a potential role of CENP-N-DNA interaction in CENP-A nucleosome recognition. Although the crystal structures of human CENP-A nucleosome core particle (NCP) and yeast Iml3 (CENP-L ortholog) in complex with the C-terminal domain of Chl4 (CENP-N ortholog) are available, the structure of the N-terminal domain of CENP-N (CENP-N^N^) and the pattern of interaction between CENP-N and CENP-A NCP remain elusive, significantly limiting our understanding of CENP-N recognition of CENP-A NCP at kinetochores.

**Figure 1:**
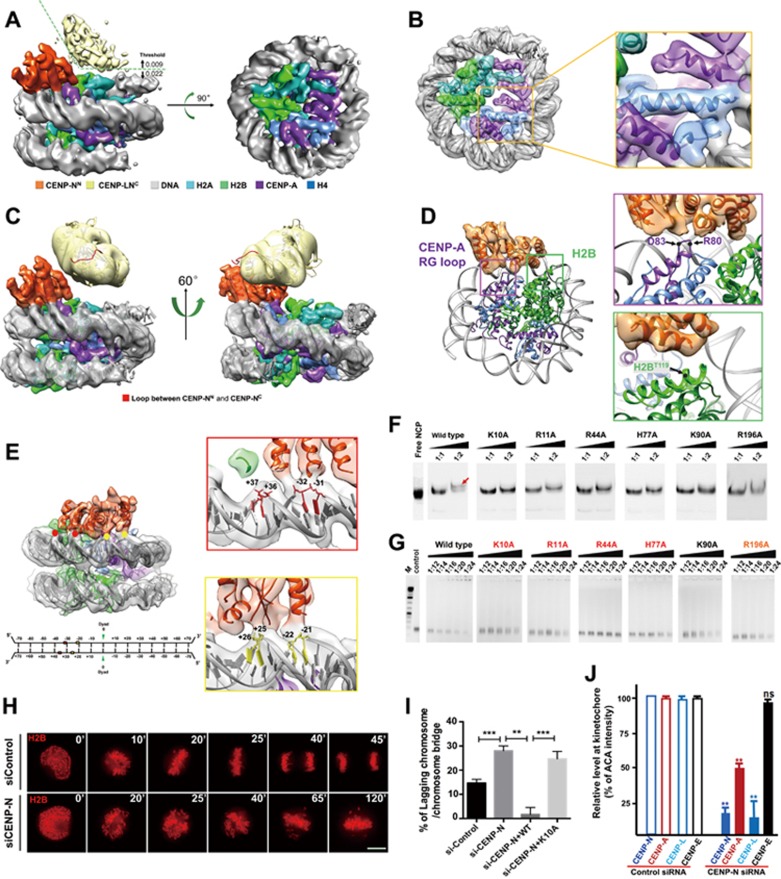
Structural insights into the recognition of CENP-A nucleosomes by CENP-N via CENP-A and nucleosomal DNA. **(A-C)** Overall cryo-EM structure of CENP-A NCP/CENP-LN complex. **(A)** Surface views of the cryo-EM structure of CENP-A NCP/CENP-LN. **(B)** A composite CENP-A NCP atomic model consisting of the H2A/H2B/CENP-A/H4 octamer and 147 bp DNA was docked into the cryo-EM density map. The map was segmented and colored orange for CENP-N^N^, yellow for CENP-LN^C^, gray for DNA, cyan for H2A, green for H2B, purple for CENP-A, and blue for H4, respectively. **(C)** The helices of CENP-N^N^ and the heterodimeric structure of yeast CENP-LN^C^ were docked into the density and colored consistently. The loop region between CENP-N^N^ and CENP-LN^C^ is colored red. **(D)** Molecular basis for the recognition of CENP-A RG loop and H2B C-terminus by CENP-N. The left panel shows the overall view of the interaction, and the RG loop of CENP-A and the T119 of H2B are labeled purple and green, respectively. The conserved R80 and D83 on CENP-A and the T119 on H2B shown in right zoom-in windows are indicated with black dots. **(E)** Structural basis for the interaction between nucleosomal DNA and CENP-N. Overall view of the interaction is shown left, and four contact sites are indicated by red and yellow dots. Details of the interaction of CENP-N with −32/−31 nt and +37/+36 nt groups, and −22/−21 nt and +26/+25 nt groups are shown in the right zoom-in windows. Schematic representation of 147 bp 'Widom 601' DNA sequence used in nucleosome reconstitution is shown below. **(F)** Gel shift assays for the interaction between CENP-A NCP and wild-type CENP-N^1-214^ or its mutants. NCP:CENP-N molar ratio, 1:1 and 1:2. The slow migrating band indicates that the CENP-A NCP binds CENP-N^1-214^ (indicated by red arrow). Free NCP was loaded to the gel as control. **(G)** Gel shift assays for the interaction between 147 bp DNA (the same DNA used for NCP reconstitution) and wild-type CENP-N^1-214^ or its mutants. A fixed amount of DNA (0.37 μM, 1 μL) was incubated with wild-type CENP-N^1-214^ or its mutants with a series of DNA:protein molar ratios of 1:12, 1:14, 1:16, 1:20 and 1:24 at 4 °C for 30 min. Residues important for DNA binding (K10, R11, R44 and H77) are labeled in red, R196 whose mutation slightly weakens the interaction is labeled in orange, K90A that has the comparable DNA binding affinity to wild type is labeled in black. M: DNA marker, control: 1 μL of 0.37 μM DNA fragment. **(H)** Representative mitotic phenotypes in HeLa cells expressing CENP-N or control siRNA shown by time-lapse microscopy, visualized with mCherry-H2B. Scale bar, 10 μm. **(I)** Quantification of chromosome segregation defects of live HeLa cells expressing control (*n* = 30) or CENP-N siRNA (*n* = 32), or coexpressing CENP-N siRNA with RNAi-resistant GFP-CENP-N-WT (*n* = 29) or GFP-CENP-N-K10A (*n* = 31). Cells exhibiting lagging chromosome or chromosome bridge were quantified. Data represent mean ± SEM from three independent experiments. Statistical significance was tested by two-sided *t*-test; ^**^P < 0.01; ^***^P < 0.001. **(J)** Quantification of relative fluorescence intensity of centromere proteins to ACA (anti-centromere antibodies) in HeLa cells expressing control or CENP-N siRNA. A total of 100 kinetochores were examined from three independent experiments. Data represent mean ± SEM from three independent experiments. Statistical significance was tested by two-sided *t*-test; ^**^P < 0.01; n.s. (non-significant) indicates *P* > 0.05.

To elucidate the molecular basis of the specific recognition of CENP-A NCP by the CENP-LN complex, we reconstituted a complex containing CENP-A NCP wrapped with 147 bp DNA and CENP-LN complex, and carried out structural studies by cryo-electron microscopy (cryo-EM) (Supplementary information, Figures S1A, S1B, S1C, S1E and S2A-S2B). By combining 130 598 particle images from 6 312 electron-counting movies recorded in a TF20 and a Titan Krios cryo-electron microscope, we obtained a cryo-EM structure of CENP-A NCP/CENP-LN complex at an average resolution of 5.8 Å (with the resolution reaching 4.5 Å in some core regions) by single-particle analysis (Supplementary information, Figures S3A, S3B, S3C, S3D, S3E, S3I and S4). Surface views of the cryo-EM structure show structural features characteristic of NCP, including well-resolved major and minor grooves of the dsDNA (Figure 1A), helices and connecting loops of the four histone proteins in CENP-A NCP (Figure 1B). Because the crystal structure of CENP-A NCP^8^ (PDB ID: 3AN2) contains only 121 bp DNA and that of the NCP with CENP-C peptide^9^ (PDB ID: 4X23) has the same length of DNA used in our study, we fitted a composite CENP-A NCP atomic model consisting of the H2A/H2B/CENP-A/H4 octamer and 147 bp DNA in the two known crystal structures into our cryo-EM density map and obtained excellent match (Figure 1B). This fitting also shows that the 13 bp DNA at the entrance and exit of CENP-A NCP missing in the CENP-A NCP crystal structure is resolved in our structure and exhibits almost the same arrangement as that of canonical H3 nucleosome (Supplementary information, Figure S5).

Attached to one face of the NCP are two extra regions of prominent densities — one proximal to and the other distal from the NCP — that are contributed by the bound CENP-LN complex (Figure 1A and 1C). The NCP-proximal density has extensive contacts with the NCP (Figure 1A), whereas the NCP-distal density has no interaction with NCP and is connected to the NCP-proximal density flexibly with one tenuous/weak density (Figure 1C). The crystal structure of yeast Chl4^C^/Iml3 (PDB ID: 4JE3) can be placed into the envelope of the NCP-distal density with the N-terminal linker of Chl4^C^ positioned at the tenuous density between the NCP-proximal and -distal regions (Figure 1C, yellow), suggesting that the extra NCP-proximal density (Figure 1C, orange) likely corresponds to CENP-N's N-terminal domain (CENP-N^N^). Indeed, gel shift assays confirmed that the N-terminal segment (aa 1-214) of CENP-N is sufficient to bind CENP-A nucleosome and free 147 bp DNA (Supplementary information, Figure S6A, S6B, S6C, S6C, S6D). Our cryo-EM map of CENP-N^N^ contains five long (∼20 Å) and 1-3 short (∼13 Å) α-helices surrounding a central β sheet with well-defined densities, especially those at the interface between CENP-N and the NCP (Figure 1A and 1C). These well-resolved secondary structures account for most of the predicted α helices and β strands (∼200 aa, see Supplementary information, Data S1). Docking this crude model of CENP-N^N^, the composite atomic model of CENP-A NCP and crystal structure of heterodimer of CENP-L with the C-terminal CENP-N (CENP-LN^C^) into our cryo-EM structure reveals numerous interactions between CENP-N and CENP-A NCP for the first time, allowing us to describe how the N- and C-terminal domains of CENP-N pack together to bridge CENP-A NCP and CENP-L.

The interface between CENP-A NCP and CENP-N^N^ covers both the histones (Figure 1D) and the adjacent nucleosomal DNA (Figure 1E). In addition to the previous reports on RG loop of CENP-A, the current cryo-EM structure offers structural insights into its function where the RG loop interacts with at least two different regions of CENP-N, one of which is located at the end of a helix (Figure 1D). Besides the conserved residue R80, D83 also appears to interact with CENP-N according to the structure (Figure 1D). In addition, residues H109-K120 from the C-terminal helix of H2B have been identified as the potential binding region for CENP-N by chemical crosslinking coupled with mass spectrometry (XL-MS)^10^. Our structure indicates that H2B^T119^is the most possible residue that is involved in CENP-N binding within this region (Figure 1D).

Compared to the RG loop of CENP-A, the contribution of the nucleosomal DNA for the binding of CENP-N has rarely been studied. To our surprise, the contacts between CENP-N and DNA occupies more than half of the interface (Figure 1E). CENP-N interacts with the phosphate backbones along two successive minor grooves (with one major groove in between). The interacting phosphate backbones on nucleosomal DNA can be divided into four groups, +37/+36 nt, −32/−31 nt, +26/+25 nt and −22/−21 nt (Figure 1E). These four DNA sites interact with four distinct regions of CENP-N to form a large DNA-protein interface, indicating an important role of CENP-N-DNA interaction in the formation and stabilization of CENP-A NCP/CENP-LN complex (Figure 1E).

Although sequence alignment reveals the conservation of RG loop motif, the lengths and/or sequences of RG loop differ in CENP-A from different species (Supplementary information, Figure S7A, S7B, S7C). Given that the specific recognition of CENP-A nucleosome by CENP-N is functionally conserved across these species, it is likely that the CENP-N from different species undergoes corresponding evolution to match the diverse RG loops of CENP-A. In contrast, CENP-N recognizes nucleosomal DNA in a sequence-independent manner by interacting with the phosphate backbones, suggesting an essential role of the positively-charged residues of CENP-N in DNA binding. To evaluate the effects of nucleosomal DNA binding by CENP-N on recognition of CENP-A NCP, we first generated several mutants of CENP-N^1-214^ by changing the conserved positively-charged amino acids to alanine based on the sequence alignment analysis of CENP-N orthologues from different species (Supplementary information, Figures S8 and S9). As shown in Figure 1F, mutations of K10, R11, R44, H77, K90 and R196 significantly reduced the CENP-A NCP binding affinity to CENP-N. Among these mutants, K10A, R11A, R44A and H77A showed obviously lower DNA binding ability, R196A slightly reduces the DNA interaction, whereas the DNA binding affinity of K90A is comparable to that of wild type (Figure 1G and Supplementary information, Figure S10). These findings indicate that residues K10, R11, R44 and H77 of CENP-N are critical for CENP-N binding to CENP-A NCP by directly interacting with nucleosomal DNA and are likely located at the interface between nucleosomal DNA and CENP-N.

To investigate the function of CENP-N in the kinetochore, we introduced siRNA oligonucleotides against CENP-N into HeLa cells and examined mitotic progression using time-lapse microscopy. As shown in Figure 1H, suppression of CENP-N resulted in a high frequency of chromosome segregation defects, including chromosome misalignment and chromosome bridges. The defects seen in the CENP-N-deficient cells were largely rescued by an RNAi-resistant wild-type CENP-N, but not K10A, a DNA binding-deficient mutant of CENP-N, demonstrating the role of the DNA binding activity of CENP-N in mitotic chromosome movements (Figure 1I and Supplementary information, Figure S11). Careful analyses of CENP-N-suppressed HeLa cells revealed that CENP-N is essential for stable centromere localization of CENP-A and CENP-L but not CENP-E (Figure 1J and Supplementary information, Figure S12).

Taken together, our study sheds lights on how CENP-N specifically recognizes CENP-A-containing chromatin and recruits CENP-L for kinetochore assembly. Recognition requires interactions of CENP-N with CENP-A RG loop, histone H2B α_C_ helix and nucleosomal DNA. The RG loop is a unique marker in CENP-A Targeting Domain (CATD) discriminating CENP-A from H3 NCP, and is specifically recognized by CENP-N. The interactions of CENP-A RG loop and H2B α_C_ helix with CENP-N^N^ observed in our structure ensure CENP-N to bind on the CENP-A NCP surface at proper orientation, consistent with the significantly impaired interaction observed for mutations of R80-G81^3^. In addition, we observed that the nucleosomal DNA makes a dominant contribution to stabilize the complex by associating with CENP-N via electrostatic interactions. Mutations of the key residues in CENP-N essential for DNA binding significantly reduced the interaction between CENP-N and CENP-A NCP *in vitro* and kinetochore localization of CENP-N *in vivo*. Given the fact that CENP-N is required for stable association of CENP-A and CENP-L but not CENP-E with the kinetochore, we reason that human kinetochores have evolved an elaborate kinetochore assembly machinery to ensure accurate chromosome segregation during cell division.
