# Supplementary material for: Molecular basis for CENP-N recognition of CENP-A nucleosome on the human kinetochore
Source: Cell Res. 2018 Jan 19;28(3):374–8. doi: 10.1038/cr.2018.13 (PMC5835772; doi:10.1038/cr.2018.13)
Supplement: Supplementary information — Data S1, Table S1 and Figures S1–S12 [file cr201813x1.pdf]

Supplementary information, Data S1 Methods and materials

Plasmids

The genes encoding full-length human H2A-H2B and CENP-A-H4 were cloned into pET28K and pETDuet-1 vector to yield $(His)_6$-H2A-H2B/pET28K and $(His)_6$-CENP-A-H4/pETDuet-1 plasmids, respectively. The $(His)_8$-CENP-N (CENP-N truncations and CENP-$N^{1\text{-}214}$ mutants) plasmids were constructed by cloning CENP-N into pET-28a (+) vector. The full-length CENP-N and CENP-L were cloned into Multi-Bac vector[1] pACEBac1 (Invitrogen) with a $(His)_6$-tag fused to its C-terminus and pIDC, respectively. The two plasmids were then fused by Cre-LoxP reaction for the expression of CENP-LN complex. For *in vivo* experiments, we cloned the full-length CENP-N into pEGFP-C2-Linker. Point mutants in CENP-N were generated with a site-directed mutagenesis polymerase Q5 (NEB). All plasmids were sequenced for verification.

Protein production

The histone plasmids were co-transformed into *E. coli* Rosetta2 (DE3) cells and plated on agar containing 50 μg/mL kanamycin and 100 μg/mL ampicillin followed by grown overnight at 37 °C. Picked colonies were inoculated into 5 mL starter culture of Luria-Bertani (LB) medium and grown with shaking for 3 hours at 37 °C. The starter culture was transferred into 50 mL LB medium and incubated at 37 °C for another 3 hours. Fifteen-milliliter of the second culture was transferred into 1L LB medium and incubated at 37 °C to an OD_{600} of ~0.5. The culture was induced with 0.4 mM isopropyl β-D-1-thiogalactopyranoside (IPTG) and incubated at 37 °C for 12~16 h. Cells were harvested and lysed using high pressure cracker in lysis buffer A (20 mM Tris-HCl, 2 M NaCl, 1 mM β-ME, pH 8.0). The human H2A/H2B/CENP-A/H4 octamer

was first purified by nickel-nitriloacetic acid (Ni-NTA) affinity chromatography [2], and the $(His)_8$-tag was cleaved by TEV protease. The histone octamer was further purified using a HiLoad Superdex 200 (16/60) (GE Healthcare) column with SEC buffer 1 (20 mM Tris, 2 M NaCl, 1 mM DTT, pH 8.0), and the peaks corresponding to histone octamer were assessed by SDS-PAGE. Purified histone octamers were wrapped with 147 base pair 'Widom 601' DNA fragment to reconstitute CENP-A NCP by dialysis [3]. The reconstituted NCP was further purified by 60 mL 5% Native-PAGE in Model 491 Prep Cell (Bio-Rad Laboratories) according to the instruction manual in 0.2 x TBE at 10 °C and at a power of 5 W running for 4 hours. Using TCS buffer (20mM Tris-HCl, 1mM EDTA, 1mM DTT, pH7.5) as an elution buffer. The peak fractions measured by UV detector at 254nm were analyzed by 15% SDS-PAGE and 5% Native-PAGE (Figure S1A-S1C).

The plasmids of CENP-N truncations or mutants were transformed into *E. coli* BL21(*DE3*) RIL (Novagen) cells and grown overnight at 37 °C in a 50 mL starter culture of Luria-Bertani (LB) media containing 50 μg/mL kanamycin. Ten milliliter overnight starter culture was transferred into 1 L LB medium and incubated at 37 °C to an OD_{600} of ~0.6–0.8. The culture was induced with 0.4 mM IPTG and incubated for ~20 hours at 16 °C. Cells were harvested by centrifugation and lysed using high pressure cracker in a lysis buffer B (50 mM Tris-HCl, 500 mM NaCl, 5 mM Imidazole, 5% Glycerol, pH 7.5). The N-terminal $(His)_8$-tagged protein was first purified by nickel-nitriloacetic acid (Ni-NTA) affinity chromatography and further purified using a Superdex 200 (10/300) increase (GE Healthcare) column with SEC buffer 2 (20 mM Tris, 500 mM NaCl, 2% glycerol, 1 mM DTT, pH 7.5), and the peaks corresponding to CENP-N were assessed by 15% SDS-PAGE (Figure S9).

The CENP-LN complex was expressed in Sf21 insect cells for 48 hours and purified in the same way as CENP-N truncations (Figure S1D-S1E).

The CENP-A NCP/CENP-LN complex was obtained by mixing purified CENP-A NCP and CENP-LN complex with a 1:1.5 molar ratio in SEC buffer 3 (20mM Tris-HCl, 50 mM NaCl, 1mM DTT, pH7.5) at 4 °C for 30 minutes followed by gel filtration with a Superose6 (10/300) (GE Healthcare) column. The peaks corresponding to CENP-A NCP/CENP-LN complex was assessed by 5% Native-PAGE and 15% SDS-PAGE and concentrated for cryo-EM grid preparation (Figure S2).

EM grid preparation and data collection

For cryo-EM sample preparation, an aliquot of 2.5μl fresh sample of concentration 0.2~0.3 mg/ml (measured by SAM 4000 at 260 nm)was applied to a glow-discharged holey carbon grid (Quantifoil, R1.2/1.3, 300 mesh). The grid was blotted for 6s under 100% humidity at room temperature (20 °C), and plunged into liquid ethane cooled by liquid nitrogen with an FEI Vitrobot (FEI Company). The grids were evaluated in an FEI Tecnai F20 (TF20) microscopy operating at 200 kV with an FEI Eagle's camera. Good grids were stored in a liquid nitrogen dewar for high resolution imaging.

A combination of TF20 and Titan Krios cryo electron microscopes, both equipped with a Gatan K2 Summit camera, were used for data collection. For the TF20 dataset, movies were acquired by *SerialEM*[4] in counting mode with an under-focus range of 1.2~2.5 μm and nominal magnification of 29,000×, corresponding to a pixel size of 1.25 Å at the specimen level. Each movie was dose-fractionated to 40 frames with 0.15s exposure time per frame. With a dose rate of ~12 counts per physical pixel per second, the total dose was ~46 electrons/Å^2. For Titan

Krios, the cryo-EM data were collected by *Leginon*[5] with the under-focus range of 1.5~2.5μm and at a nominal magnification of 130, 000× in super resolution mode, corresponding to a pixel size of 0.535 Å on the sample level. The dose rate was set to be 7 electrons per physical pixel per second and the total exposure time for each movie was 9 s, fractioned to 45 frames (200 ms per frame), resulting in a total dose of 55 electrons/Å^2.

Image processing

The TF20 and Titan datasets were separately processed at beginning and combined for the final refinement. For TF20 data, movie frames were motion-corrected by *MotionCorr2*[6] program, generating two datasets summing from all frames or frame 2-21, respectively. The former dataset was used for Contrast transfer function (CTF) estimation, particle picking and initial two-dimensional (2D) classifications, while the latter for further 2D and three dimensional (3D) analysis. *Gctf*[7] was employed to determine defocus parameters. Motion-corrected micrographs were evaluated and selected according to the ice and drift conditions. Particle picking and the following 2D and 3D classifications and refinements were all carried out in *Relion2.0*[8, 9]. After particle sorting and two rounds of 2D classifications, 583,653 particles from 3,250 micrographs were subjected to 3D analysis. Cryo-EM structure of the nucleosome containing H2B-K34Ub[10] was low-pass filtered to 50 Å and used as the initial model for 3D classifications and refinements. A round of 3D classification into 6 classes yielded 2 reasonable reconstructions (class 3 and class 4, 124,103 and 128,042 particles respectively). Refinement of class 3 generated a partially unpacked CENP-A NCP/CENP-LN complex at 7.5 Å (not shown), while class 4 yielded an intact reconstruction of the complex at an average resolution of 6.4 Å.

To improve the resolution of the interfaces, focused refinement on CENP-LN density was carried out. The additional 3D classification yielded 2 good classes with better density for CENP-N. The corresponding 79,797 particles were refined and an improved reconstruction was obtained, at an average resolution of 6.2 Å according to the "gold-standard" Fourier shell correlation (FSC) 0.143 criterion. Local resolution was estimated with *ResMap*[11].

For the Titan Krios dataset, frames were motion-corrected and binned over 2 × 2 by *MotionCorr2*, generating summed or dose weighted micrographs with a pixel size of 1.07 Å. Summed micrographs were used for CTF estimation, particle picking and initial 2D classification, and dose weighted data for further 2D and 3D classifications or refinements. CTF parameters was estimated by *ctffind4*[12]. Micrographs were manually evaluated and those with ice contamination or severe drift were discarded. All subsequent analysis was performed with *Relion2.1*[8, 9]. Due to the preferred orientation distribution, two rounds of particle picking were carried out using different templates that facilitated top view and side-view particle picking respectively. After particle sorting and 2D classifications 88,227 top view and 136,077 side view particles from 3,062 micrographs were qualified for further 3D analysis. A round of 3D classification into 6 classes with TF20 CENP-A NCP/CENP-LN complex reconstruction as the starting model (low pass filtered to 40 Å) yielded a promising intact complex structure (class 5, refined from 50,801 particles). The subsequent refinement reached an average resolution of 5.6 Å.

In the end the two datasets were combined to generate the final reconstruction. TF20 particles was rescaled in Fourier space to a pixel size of 1.07 Å, based on the calibrated pixel size by calculating the final reconstructions' cross-correlation coefficients in *UCSF Chimera*[13]. Masked

refinement of merged particles generated the final reconstruction with improved resolution for CENP-N.

The final resolution was reported 5.8 Å based on the 0.143 FSC criterion. Local resolution was estimated by *ResMap*[11]. The 5.8 Å structure was analyzed and interpreted in this paper.

Modelling

The atomic models of Widom 601 DNA (PDB ID: 4X23)[14], H2A/H2B/CENP-A/H4 octamer histones (PDB ID: 3AN2)[15] were fitted into the 3D density maps using *UCSF Chimera*[13], yielding a composite atomic model for the CENP-A NCP.

Five long (20 Å) and 1~3 short (13 Å) helices and a central β sheet were clearly resolved in our cryo-EM map of CENP-N^{N} (orange in Figure 1A and C). These secondary structures correspond to secondary structures predicted by *PSIPRED*[16] based on the amino-acid sequence of CENP-N^{N}. A polyalanine helix model was built with *Coot*[17] based on each helix density resolved in our cryo-EM map of CENP-N^{N}. The NCP-distal density has no interaction with NCP was docked with yeast Chl4^{C}/Iml3 (PDB ID: 4JE3)[18] corresponding with the CENP-LN^{C}. All figures were prepared with *UCSF Chimera*.

Gel shift assays

The interactions of CENP-LN or CENP-N truncations and mutants with CENP-A NCP were analyzed by 5% Native-PAGE gel electrophoresis. Three microliters of CENP-A NCP (~0.77 μM) was mixed with different amounts of CENP-LN (Figure S2A), CENP-N truncations (Figure S6A-S6C) or mutants (Figure 1F) in SEC buffer 3 for 30~60 minutes at 4 °C prior to Native-PAGE gel electrophoresis.

The interactions between CENP-N truncations and mutants with 147 bp DNA were analyzed by 1.5% native agarose gel electrophoresis (Figure 1G) or 5% Native-PAGE gel electrophoresis (Figure S6D). One microliter (Figure 1G) or four microliters (Figure S6D) of 0.37 μM 147 bp DNA were incubated with CENP-N^{1-214} or its mutants in different amounts for 30 minutes at 4 °C in 20 mM Tirs-HCl, 350 mM NaCl, 1 mM DTT, pH 7.5.

Cell culture, synchronization and transfection

HeLa cells, from American Tissue Culture Collection, were maintained as subconfluent monolayers in Dulbecco's modified Eagle's medium (DMEM, Gibco) with 10% (vol/vol) fetal bovine serum (FBS; Hyclone) and 100 units/mL penicillin plus 100 μg/mL streptomycin (Gibco) at 37 °C with 5% CO_2. For cell synchronization, aliquots of HeLa cells were synchronized at G1/S with 2.5 mM thymidine (Sigma-Aldrich) for 16 h, washed with PBS three times, and then cultured in thymidine-free medium for appropriate time intervals.

siRNA

CENP-N siRNAs (Dharmacon, M-015872-02-0005) were used according to the manufacturer's instructions. Buffer alone was used in control experiments. All the siRNAs or constructs were transfected into HeLa cells with Lipofectamine 3000 (Invitrogen) following the manufacturer's protocol.

Antibodies

For immunofluorescence assay, the following antibodies were used:, ACA (anti-centromere antibodies, gift from Don W. Cleveland, University of California at San Diego), rabbit anti-CENP-A (2186, Cell Signaling Technology), rabbit anti-CENP-N (PA5-65747, Invitrogen) and rabbit anti-CENP-L (PA5-60736, Invitrogen). Antibody against CENP-E (HpX) were generated

as described previously[19, 20]. Secondary antibodies were purchased from Jackson ImmunoResearch.

Immunofluorescence and live cell imaging

HeLa cells transfected with CENP-N siRNA were rinsed for 1 min with PHEM buffer (100 mM PIPES, 20 mM HEPES, pH 6.9, 5 mM EGTA, 2 mM $MgCl_2$, and 4 M glycerol) and permeabilized for 1 min with PHEM plus 0.1% Triton X-100 as described previously[21]. Extracted cells were then fixed using PHEM buffer supplemented with 3.7% paraformaldehyde. After blocking with 1% bovine serum albumin (Sigma-Aldrich) in PBST (PBS with 0.05% Tween-20) buffer for 45 min at room temperature, the fixed cells were incubated with primary antibodies in a humidified chamber for 1 hour followed by secondary antibodies for 1 hour at room temperature. The DNA was stained with DAPI (Sigma-Aldrich). Images were acquired by DeltaVision softWoRx software (Applied Precision) and processed by deconvolution and z-stack projection.

For live cell imaging, HeLa cells transfected with CENP-N siRNA and constructs of GFP-CENP-N wild type or K10A mutant were cultured in glass-bottom culture dishes (MatTek) and maintained in CO_2-independent media (Gibco) supplemented with 10% (vol/vol) FBS and 2 mM glutamine[22]. During imaging, the dishes were placed in a sealed chamber at 37 °C. Images of living cells were taken with a DeltaVision microscopy system (Applied Precision Inc.). Image processing was performed with SoftWoRx (Applied Precision Inc.). To trace chromosomes in mitosis, frames were collected at 3-5 min intervals. Images were prepared for publication using Adobe Photoshop software.

Fluorescence intensity quantification

Quantification of fluorescence intensity of kinetochore-associated proteins was performed as described previously using ImageJ (NIH)[23]. In brief, the average pixel intensities from no less than five cells (which were randomly selected) were measured, and background pixel intensities were subtracted. The pixel intensities at each kinetochore pair were then normalized against ACA values to account for any variations in staining or image acquisition.

Statistics

Two-sided unpaired Student's *t*-test was applied for experimental comparisons, using GraphPad Prism. Differences were considered significant when $p < 0.05$.

Reference:

1 Bieniossek C, Imasaki T, Takagi Y, Berger I. MultiBac: expanding the research toolbox for multiprotein complexes. *Trends in biochemical sciences* 2012; **37**:49-57.

2 Shim Y, Duan MR, Chen X, Smerdon MJ, Min JH. Polycistronic coexpression and nondenaturing purification of histone octamers. *Analytical biochemistry* 2012; **427**:190-192.

3 Dyer PN, Edayathumangalam RS, White CL *et al.* Reconstitution of nucleosome core particles from recombinant histones and DNA. *Methods in enzymology* 2004; **375**:23-44.

4 Mastronarde DN. Automated electron microscope tomography using robust prediction of specimen movements. *J Struct Biol* 2005; **152**:36-51.

5 Suloway C, Pulokas J, Fellmann D *et al.* Automated molecular microscopy: The new Leginon system. *J Struct Biol* 2005; **151**:41-60.

6 Zheng SQ, Palovcak E, Armache JP, Verba KA, Cheng YF, Agard DA. MotionCor2: anisotropic correction of beam-induced motion for improved cryo-electron microscopy. *Nat Methods* 2017; **14**:331-332.

7 Zhang K. Gctf: Real-time CTF determination and correction. *J Struct Biol* 2016; **193**:1-12.

8 Scheres SHW. A Bayesian View on Cryo-EM Structure Determination. *J Mol Biol* 2012; **415**:406-418.

9 Kimanius D, Forsberg BO, Scheres SHW, Lindahl E. Accelerated cryo-EM structure determination with parallelisation using GPUs in RELION-2. *Elife* 2016; **5**.

10 Li JB, He QQ, Liu YT *et al.* Chemical Synthesis of K34-Ubiquitylated H2B for Nucleosome Reconstitution and Single-Particle Cryo-Electron Microscopy Structural Analysis. *Chembiochem* 2017; **18**:176-180.

11 Kucukelbir A, Sigworth FJ, Tagare HD. Quantifying the local resolution of cryo-EM density maps. *Nat Methods* 2014; **11**:63-65.

12 Rohou A, Grigorieff N. CTFFIND4: Fast and accurate defocus estimation from electron micrographs. *J Struct Biol* 2015; **192**:216-221.

13 Pettersen EF, Goddard TD, Huang CC *et al.* UCSF chimera - A visualization system for exploratory research and analysis. *J Comput Chem* 2004; **25**:1605-1612.

14 Kato H, Jiang JS, Zhou BR *et al.* A Conserved Mechanism for Centromeric Nucleosome Recognition by Centromere Protein CENP-C. *Science* 2013; **340**:1110-1113.

15 Tachiwana H, Kagawa W, Shiga T *et al.* Crystal structure of the human centromeric nucleosome containing CENP-A. *Nature* 2011; **476**:232-235.

16 McGuffin LJ, Bryson K, Jones DT. The PSIPRED protein structure prediction server. *Bioinformatics* 2000; **16**:404-405.

17 Emsley P, Cowtan K. Coot: model-building tools for molecular graphics. *Acta Crystallogr D* 2004; **60**:2126-2132.

18 Hinshaw SM, Harrison SC. An Iml3-Chl4 heterodimer links the core centromere to factors required for accurate chromosome segregation. *Cell reports* 2013; **5**:29-36.

19 Yen TJ, Li G, Schaar BT, Szilak I, Cleveland DW. CENP-E is a putative kinetochore motor that accumulates just before mitosis. *Nature* 1992; **359**:536-539.

20 Yao X, Anderson KL, Cleveland DW. The microtubule-dependent motor centromere-associated protein E (CENP-E) is an integral component of kinetochore corona fibers that link centromeres to spindle microtubules. *The Journal of cell biology* 1997; **139**:435-447.

21 Dou Z, Liu X, Wang W *et al.* Dynamic localization of Mps1 kinase to kinetochores is essential for

accurate spindle microtubule attachment. *Proceedings of the National Academy of Sciences of the United States of America* 2015; **112**:E4546-4555.

22 Ding X, Yan F, Yao P *et al.* Probing CENP-E function in chromosome dynamics using small molecule inhibitor syntelin. *Cell Res* 2010; **20**:1386-1389.

23 Mo F, Zhuang X, Liu X *et al.* Acetylation of Aurora B by TIP60 ensures accurate chromosomal segregation. *Nature chemical biology* 2016; **12**:226-232.

Supplementary Table and Figures:

Table S1: UniProt Entries of the protein sequences used for sequence alignments.

Figure	Species	UniProt Entry
**S5A**	*Homo sapiens*	P49450
	Mus musculus	O35216
	Cricetulus griseus	Q8R565
	Bos taurus	P49449
**S5B**	*Homo sapiens*	P49450
	Xenopus laevis	Q569M3
	Saccharomyces cerevisiae	P36012
	Schizosaccharomyces pombe	Q9Y812
**F**	*Homo sapiens*	Q96H22
	Mus musculus	Q9CZW2
	Rattus norvegicus	Q5U2W4
	Bos taurus	Q32LL9
	Canis lupus familiaris	F1PCB7
	Pan troglodytes	H2QBK6
	Oryctolagus cuniculus	G1SCF8
	Macaca mulatta	G7NR50
	Ovis aries	W5PED9
	Cavia porcellus	H0V272

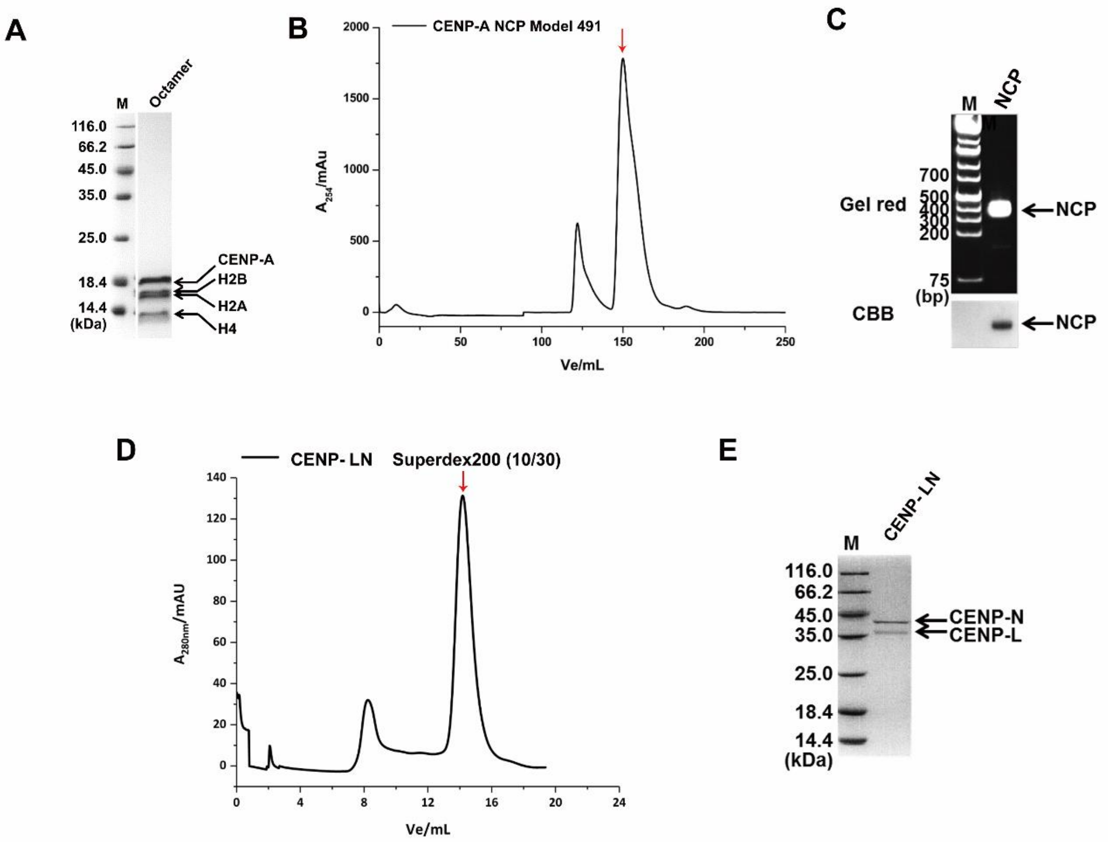

Supplementary information, Figure S1. Reconstitution of human CENP-A nucleosome core particle (NCP) and purification of CENP-LN complex.

(A) Purified $(H2A/H2B/CENP\text{-}A/H4)_2$ octamer by Ni-NTA for CENP-A NCP reconstitution.

(B-C) Purification of CENP-A NCP by 5% Native-PAGE in a Model 491 Prep Cell (Bio-Rad Laboratories (B), the purified NCP were assessed by Native-PAGE (C).

(D-E) SEC result for CENP-LN complex using Superdex200 (10/30) (GE Healthcare) column (D), the purified CENP-LN complex were assessed by SDS-PAGE (E).

The fraction peaks corresponding to CENP-A NCP and CENP-LN are indicated by red arrows.

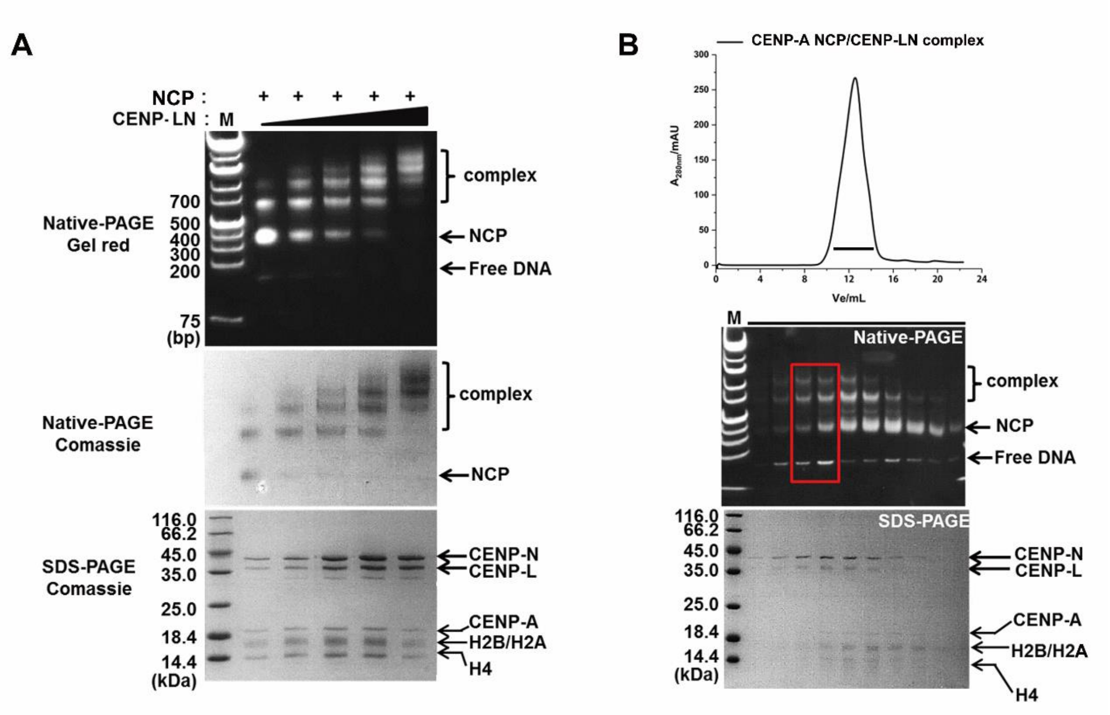

Supplementary information, Figure S2. Purification of CENP-A NCP/CENP-LN complex.

(A) Gel shift assays for the interaction between CENP-A NCP and CENP-LN complex.

(B) Purification of CENP-A NCP/CENP-LN complex by Superose6 (10/300) (GE Healthcare) column. The fractions were assessed by Native-PAGE and SDS-PAGE and the samples used for cryo-EM were indicated with red rectangle.

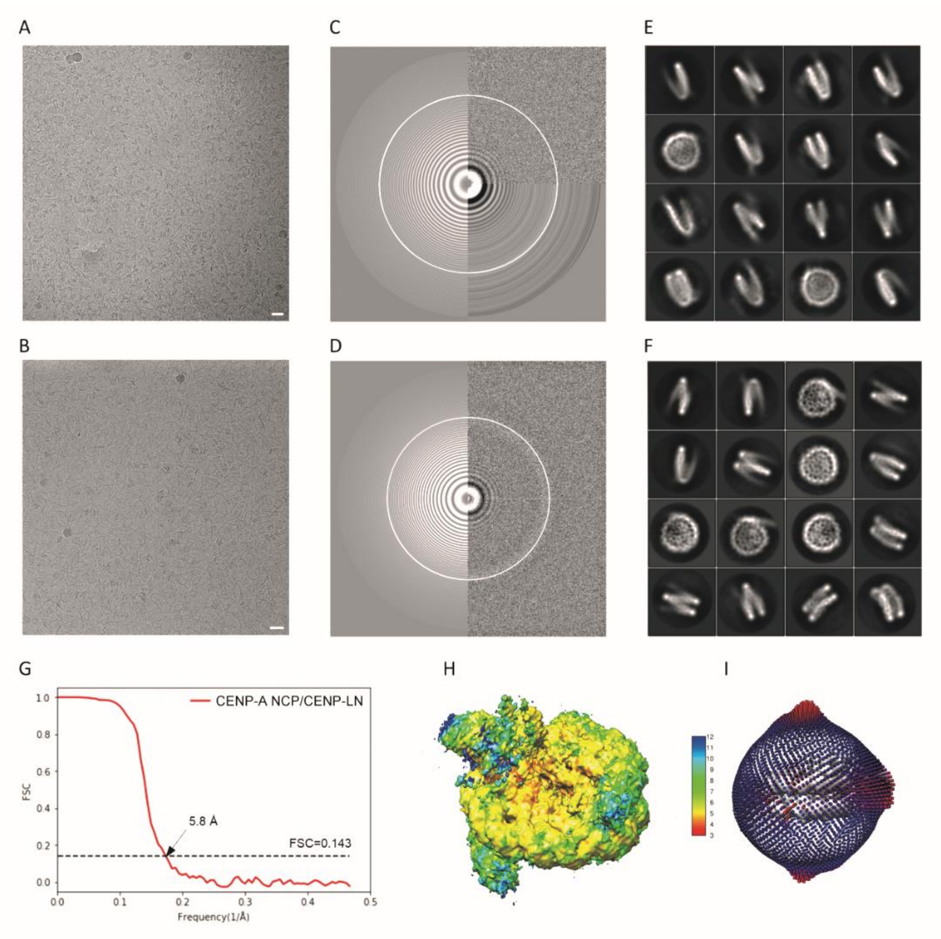

Supplementary information, Figure S3. Cryo-EM structure determination and resolution assessment of CENP-A NCP/CENP-LN complex.

(A-F) Cryo-EM structure determination**.** Representative micrograph of (A) TF20 and (B) Titan Krios cryo-EM data. (C) CTF estimation of image in (A) by *Gctf* with an estimated image resolution of 3.8 Å. (D) *Ctffind* showed Thon rings in the Fourier spectrum of the image in (B) extending to 1/3.7 $Å^{-1}$. Selected 2D class averages of the sample are shown in (E) for TF20 and (F) for Titan datasets. Scale bar in (A) and (B) is 20 nm. (G-I) Validation of CENP-A NCP/CENP-LN complex structure. (G) "Gold-standard" FSC coefficient curve of the final reconstruction showed an overall resolution of 5.8 Å. (H) Local resolution estimation by *ResMap*[11]. The core region resolution reached about 4.5 Å. (I) Orientation distribution for particles included in the final reconstruction.

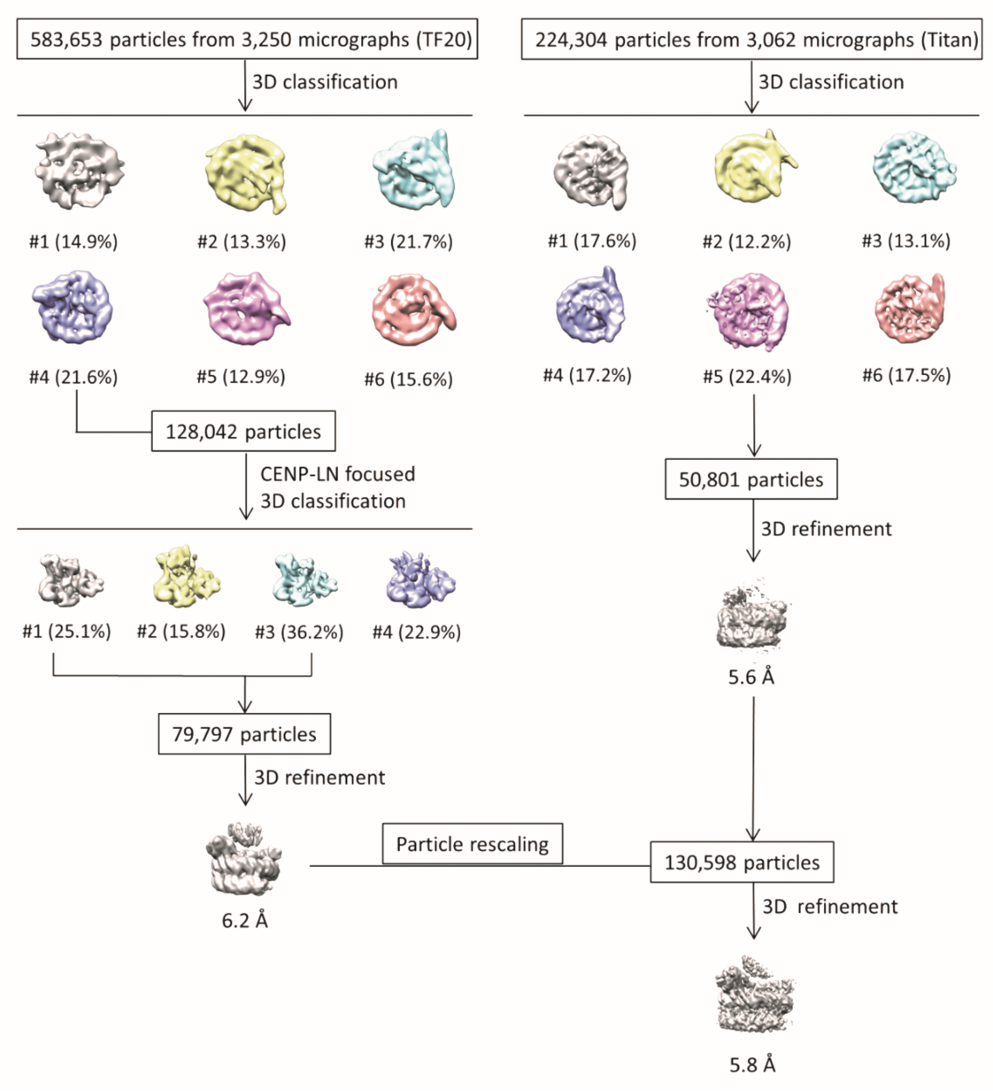

Supplementary information, Figure S4. Flow chart of cryo-EM data processing.

TF20 and Titan Krios data were first separately processed and then combined to generate the final reconstruction. For both datasets particles were picked and sorted, and subjected to 2D classifications in *Relion*. Good particles were further processed by 3D analysis. For the TF20 data an additional 3D classification that focused on CENP-LN density and skipped alignment was carried out. Before combination, particles from TF20 were rescaled to match those from the Titan Krios.

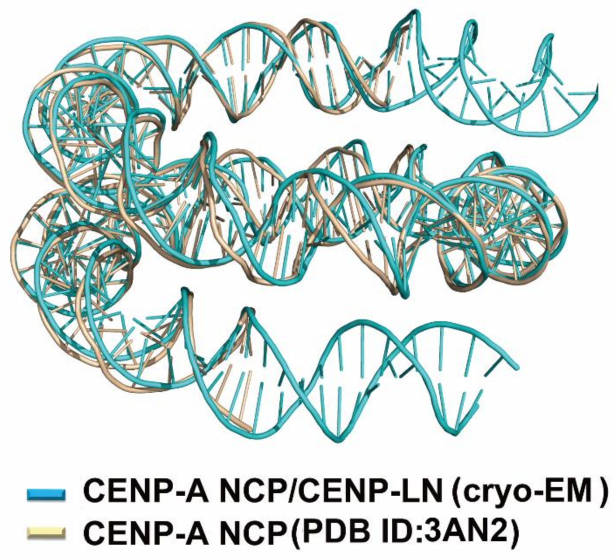

Supplementary information, Figure S5. Superposition of the necleosomal DNA (cyan) from our cryo-EM structure of CENP-A NCP/CENP-LN complex and the previously reported crystal structure of CENP-A nucleosome (wheat, PDB ID: 3AN2).

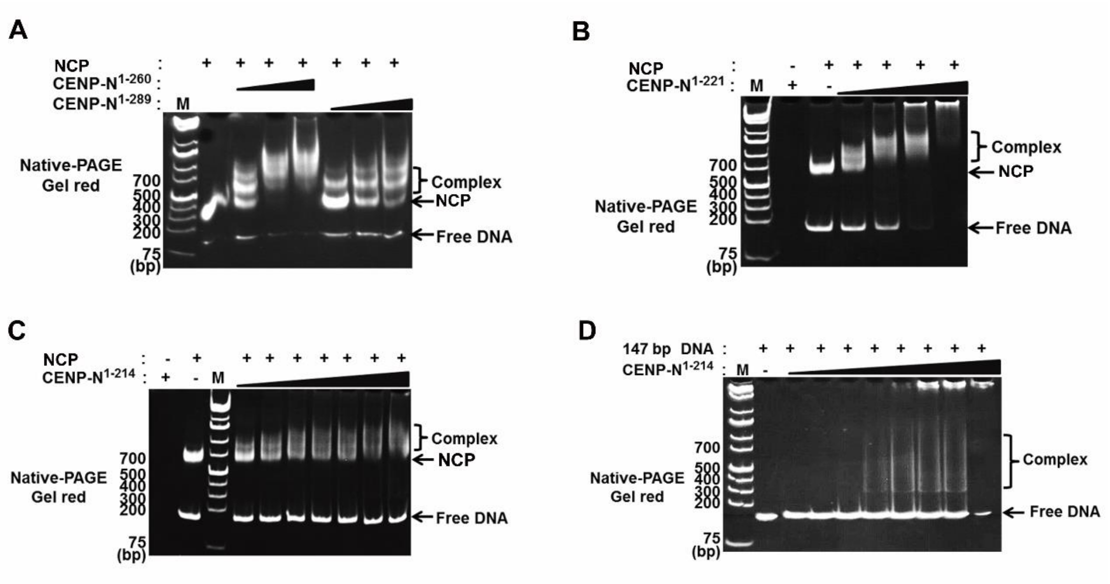

Supplementary information, Figure S6. Interactions between CENP-A NCP and CENP-N truncations.

(A-C) Native-PAGE results of the binding of CENP-A NCP by (A) CENP-$N^{1\text{-}260}$, CENP-$N^{1\text{-}289}$, (B) CENP-$N^{1\text{-}221}$ and (C) CENP-$N^{1\text{-}214}$.

(D) Native-PAGE result of the interaction between 147 bp DNA and CENP-$N^{1\text{-}214}$. The bands corresponding to free DNA, free NCP and the complex formed by NCP or DNA with CENP-N are labeled.

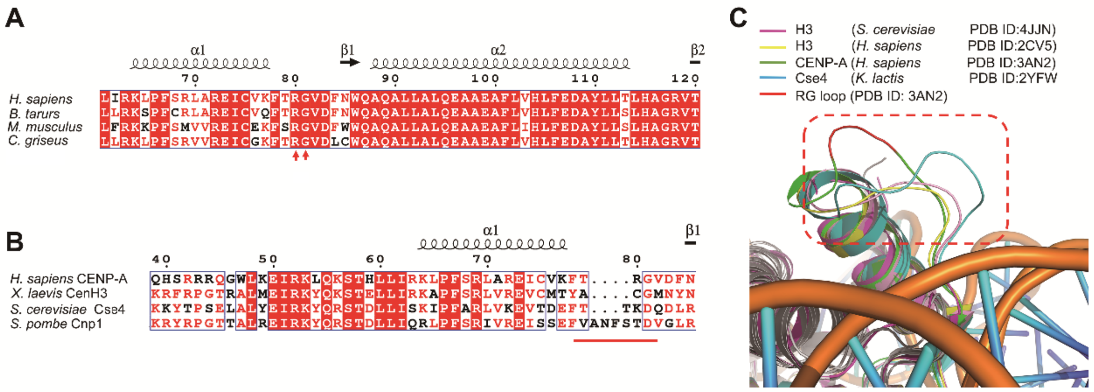

Supplementary information, Figure S7. Comparison of the sequences and structures of CENP-A RG loops from different species.

(A-B) Sequence alignments of human CENP-A against its orthologous from (A) higher mammal species, (B) *Xenopus laevis* and yeast. the RG loop region is highlighted with red arrows in (A) and red line in (B). The UniProt entries of the CENP-A orthologous used for sequence alignment are listed in Table S1.

(C) Structural comparison of the RG loop (red) in human CENP-A (green) and its corresponding regions in human H3 (yellow), *S. cerevisiae* H3 (pink) and *Kluyveromyces lactis* Cse4 (cyan).

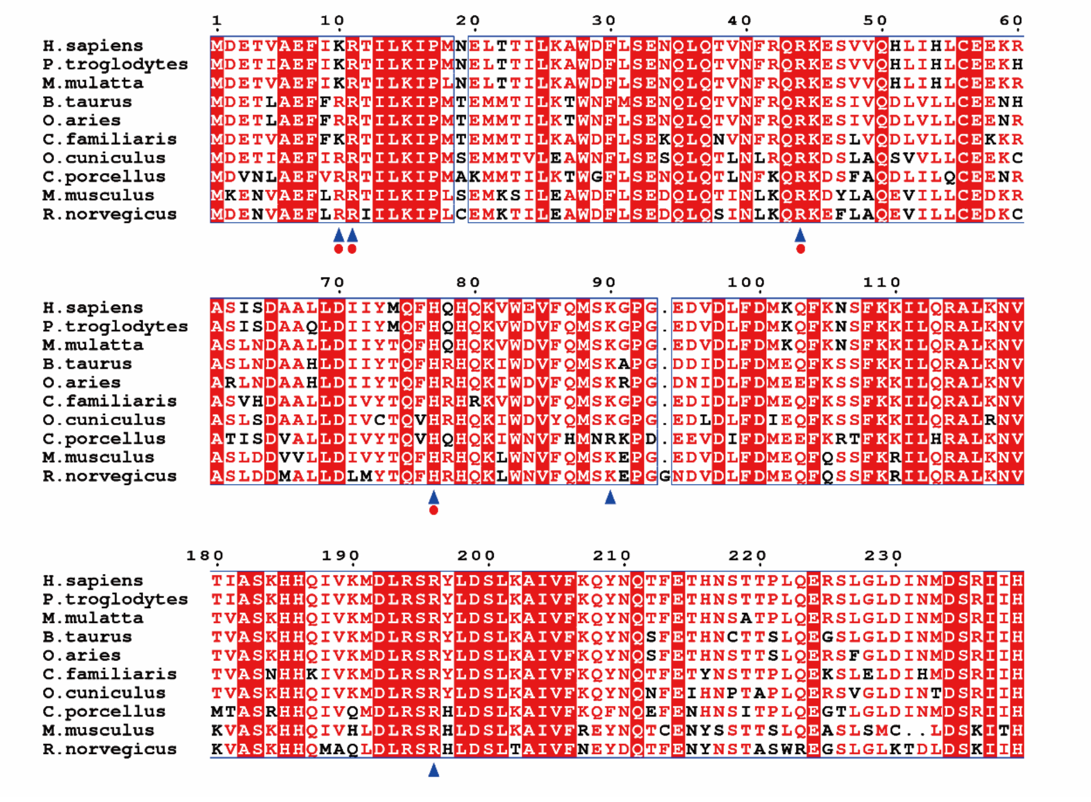

Supplementary information, Figure S8. Sequence alignments of human CENP-N against its orthologous.

The key residues identified to be essential for CENP-A NCP and necleosomal DNA binding are indicated with blue triangle and red dots, respectively. The UniProt entries of the CENP-N orthologous used for sequence alignment are listed in Table S1.

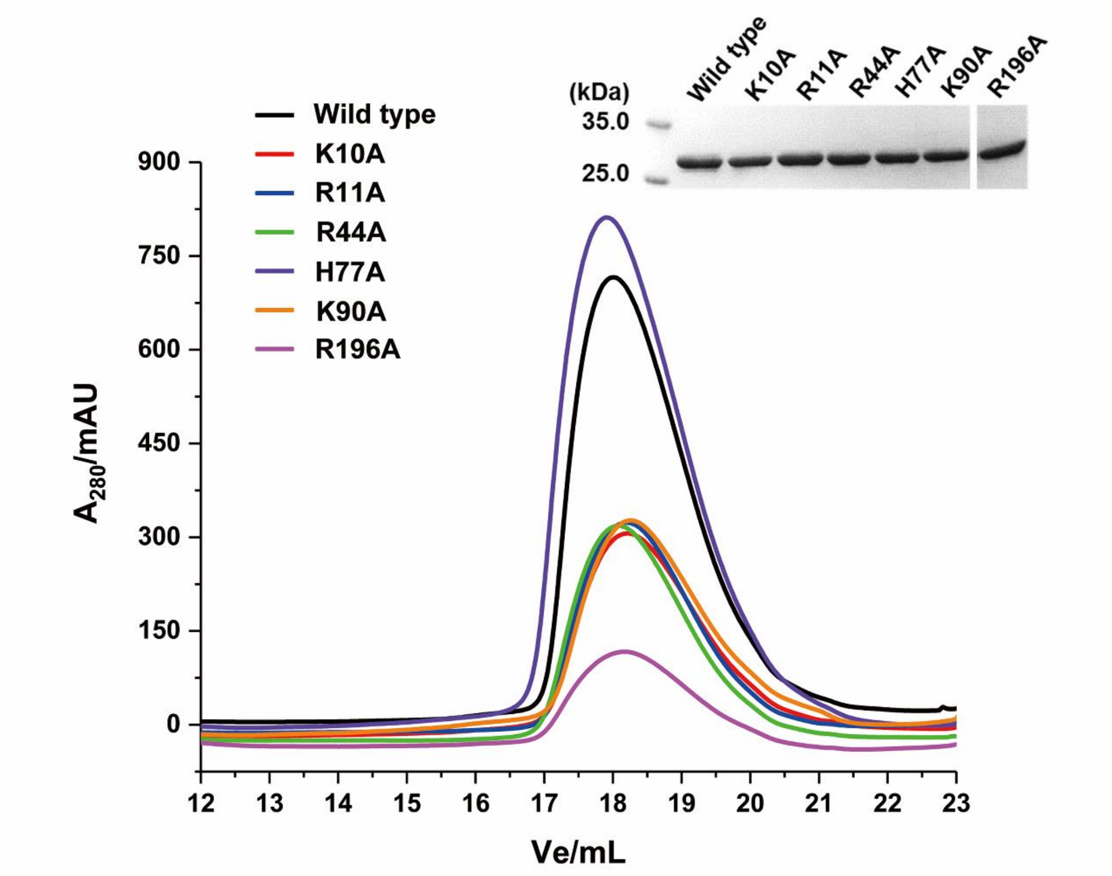

Supplementary information, Figure S9. Purification of CENP-N$^{1\text{-}214}$ variants.

Purification of wild type CENP-N$^{1\text{-}214}$ and its mutants using Superdex200 (10/300) increase (GE Healthcare) column. The peaks corresponding to CENP-N$^{1\text{-}214}$ variants were analyzed by SDS-PAGE.

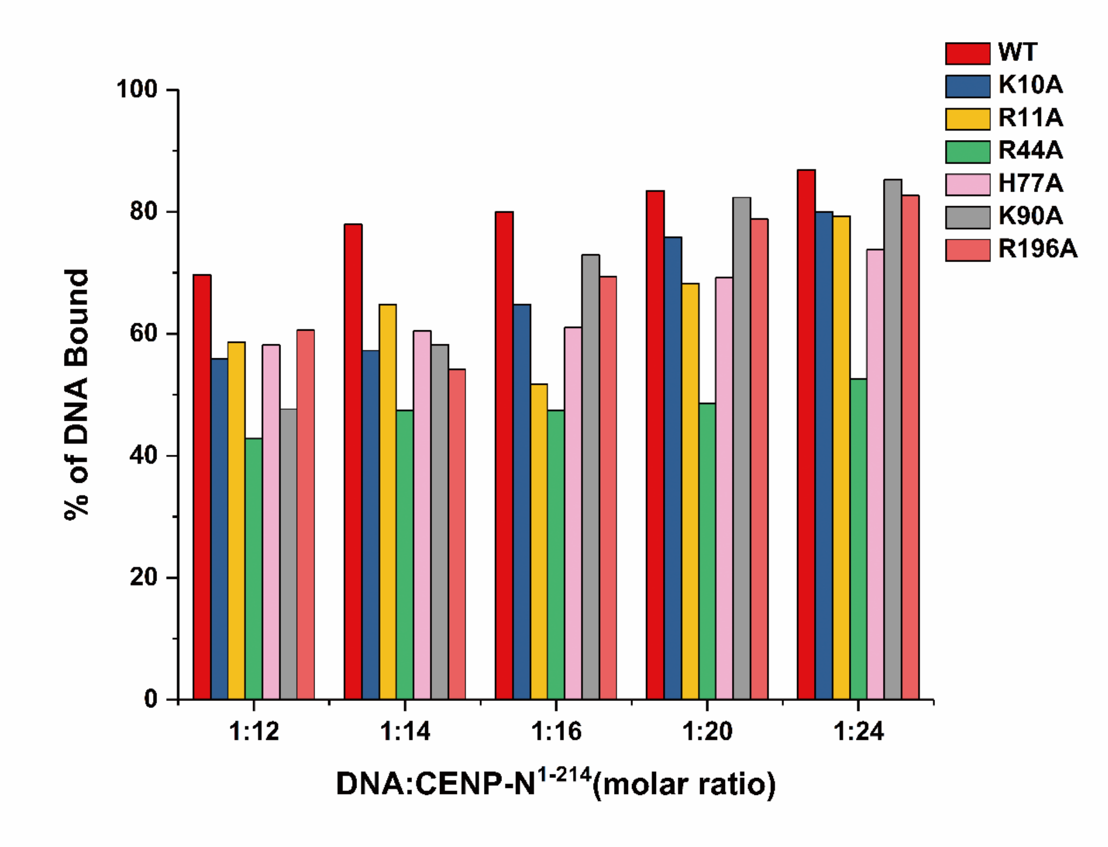

Supplementary information, Figure S10. Quantification of percentage of DNA bound to CENP-N$^{1-214}$ WT and mutants relative to Figure 1G.

The band intensity was quantified by using ImageJ (https://imagej.nih.gov/ij/index.html).

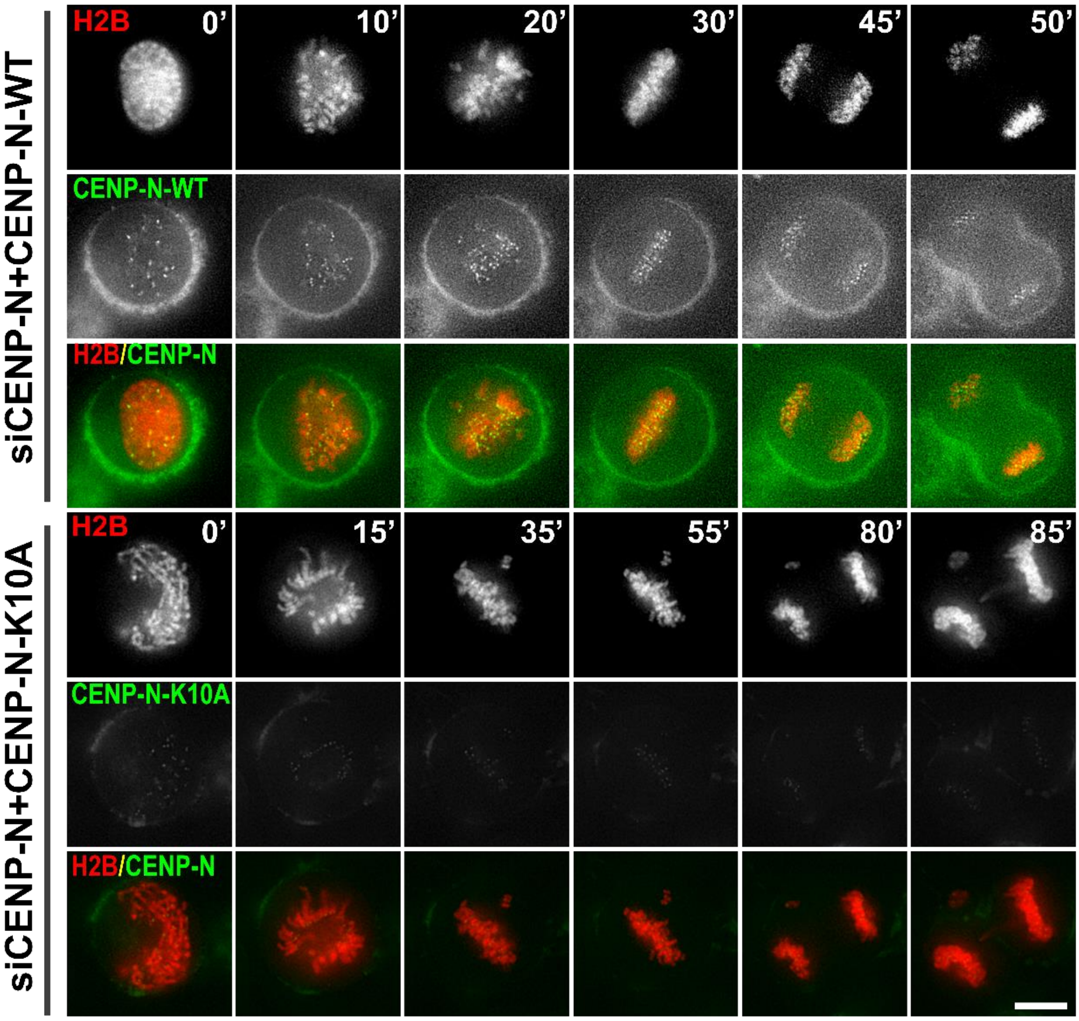

Supplementary information, Figure S11. Accurate chromosome segregation in mitosis requires CENP-N DNA-binding activity.

Representative mitotic phenotypes in HeLa cells coexpressing CENP-N siRNA and RNAi-resistant GFP-CENP-N-WT or GFP-CENP-N-K10A shown by time-lapse microscopy. Chromosomes were visualized by cotransfecting HeLa cells with mCherry-H2B. Scale bar, 10 μm.

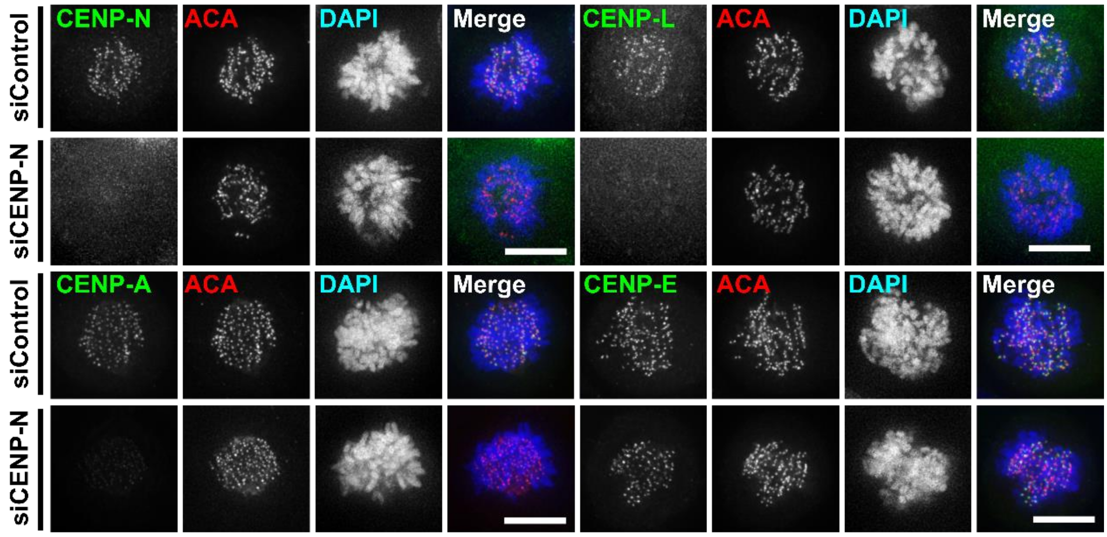

Supplementary information, Figure S12. Centromere localization of CCAN components is dependent on CENP-N.

HeLa cells expressing CENP-N siRNA were fixed and immunostained with CENP-N, CENP-L, CENP-A, CENP-E antibodies, respectively. ACA, anti-centromere antibodies. DNA was stained by DAPI. Scale bars, 10 µm.
